# Clinical Evaluation of Exercise-Induced Physiological Changes in Military Working Dogs (MWDs) Resulting from the Use or Non-Use of Cooling Vests during Training in Moderately Hot Environments

**DOI:** 10.3390/ani12182347

**Published:** 2022-09-08

**Authors:** Mila Benito, Diego Lozano, Francisco Miró

**Affiliations:** 1 Department of Animal Medicine and Surgery, Faculty of Veterinary Medicine, CEU-Cardenal Herrera University, 46115 Valencia, Spain; 2 Centro Militar de Veterinaria de la Defensa, 28024 Madrid, Spain; 3 Department of Comparative Anatomy and Pathology, University of Córdoba, 14071 Córdoba, Spain

**Keywords:** military working dogs (MWDs), cooling vests, canine sports medicine

## Abstract

**Simple Summary:**

A cooling vest is a clothing article especially designed to reduce body temperature and make exposure to heat in hot climates or environments more bearable. Such cooling vests can be of significant help to military working dogs (MWDs) in high-temperature regions. Dogs performing scent-detection tasks could benefit from the use of a cooling vest, if proven useful, by reducing the risk of heat stroke and olfactory fatigue. As different models of cooling vests are available for dogs, our aim was to compare wearing nothing versus two different models of cooling vests in a homogenous dog population during physical exercise (moderate-intensity running). We observed that the evaporative cooling waistcoat performed best. In conclusion, the waistcoats improve the cooling of the dogs during and after exercise, and differences between the two garment models exist.

**Abstract:**

Nose work with military working dogs (MWDs) in warmer-than-usual areas has led us to look for new tools to reduce both heat stress and the risk of heat stroke. One of the different strategies to manage heat stress is the use of cooling vests, such as those used in humans. The aim was to assess three cooling conditions (using two different cooling vests during exercise and the non-use of such garments) by measuring core body temperature, systemic blood pressure and pulse rate before and after the exercise (moment: four measurement times) in military dogs of the I Military Police Battalion (in Valencia, Spain). All dogs were evaluated under all three conditions during the three days of the study. Significant differences were observed between condition, moment, and the interaction of these two factors, in relation to core body temperature and pulse rate. Therefore, the use of an evaporative cooling vest may further be useful as a routine thermal control and conditioning measure in MWDs.

## 1. Introduction

The most important consequence of hyperthermia, described as an increase in body temperature above the accepted reference range [1] under conditions of elevated ambient temperature or exercise, is heat-related illness (HRI). The most important clinical signs of HRI are highly limiting for military working dogs (MWDs), as they include respiratory distress, lethargy, collapse, vomiting, diarrhea, hypersalivation, ataxia, seizures, hemorrhage, or coagulation problems, among others [2]. MWDs, in their different specialties, are confronted, together with their canine handlers, with extremely high temperature conditions, which can have a serious negative impact on the health of the dogs and the safety of the canine handlers. Although the exact number of dogs suffering from heat stress worldwide is not known, 24.8% of dogs from k9 units have been reported as traumatic deaths in service in US civilian law enforcement between 2002 and 2012 [3]. In addition, their work in olfactory detection such as Explosive Detection Dogs (EDDs) or Drug Detection Dogs (DDDs) can be seriously affected when there is an increased vulnerability to heat stress. Authors such as Robbins et al. have assessed the association of environmental and dog-specific factors on stamina, finding a direct relationship of temperature on the performance of these dogs. One of their important conclusions was that as outdoor temperature increased, stamina decreased [4]. In humans, depletion has been found to occur at the same level as the rise in internal body temperature [5]. This influence of high temperatures on stamina in working dogs, together with the fact that the selection of these dogs usually does not consider their ability to adapt to heat but other physical, fitness and attitudinal parameters specific to their tasks, makes us search for new methods of heat stress control for these valuable dogs.

There are different strategies for body temperature control in dogs, such as hydration [6,7] or the use of cooling waistcoats, created specifically to ensure the health and well-being of MWDs. One of the models of these cooling waistcoats is the so-called Phase Change Material (PCM-CV), which uses pre-cooled cooling pads and has a cooling duration of up to 4 h. The effectiveness of these cooling vest models in lowering the skin temperature and heart rate has been confirmed for human sports [8,9]. In MWDs, however, there is insufficient research available on the effects of these garments on the thermoregulation of these dogs.

The main aim of this study was to determine whether military working dogs (MWDs) could benefit from body temperature rise control using a cooling vest during exercise and in the subsequent resting phase in warm environments.

Thus, the second objective of this clinical study was to compare both types of waistcoats (PCM-CV and Ev-CV models) in a homogenous MWD canine population with the ultimate goal of selecting the most comfortable and effective cooling vest to reduce fatigue and the likelihood of muscle damage or heat stress in these dogs.

## 2. Materials and Methods

### 2.1. The Animals

This clinical study was carried out with 13 clinically healthy military working dogs (MWDs) habituated to regular olfactory work and trained in physical sport. All dogs were adults, aged between 2 and 6 years, with an average age of 4 years. Table 1 shows the data of the dogs participating in the study, of which ten were males (76.92%) and three were females (23.08%). The majoritarian breed was German Shepherd (GS), with 69%, compared to 31% for Belgian Shepherd Malinois (BSM). None of the dogs had been previously sterilized. All dogs were run through the three experimental conditions over the three days of data collection. The allocation of conditions was randomized and counterbalanced, so that the influence of a fatigue effect was controlled.

All dogs were in good physical condition, with a score of 4–5 out of a possible 9 in a body condition scoring system [10] and were acclimatized to the temperatures at which the clinical study was carried out. No treats were given at the end of daily exercise and the timing of the food did not interfere with the clinical study, since it was administered at least three hours after exercise, following the usual dogs’ habits. The dogs had freely available water before the physical effort and after the arrival measurements were taken.

All handlers put the cooling vest on their dogs in the kennel (except for the dogs that were not supposed to wear them that day). The start line was right next to the kennel, so that the dogs did not walk more than 10 m with their handlers in any case.

### 2.2. The Field Test

**Figure 1:**
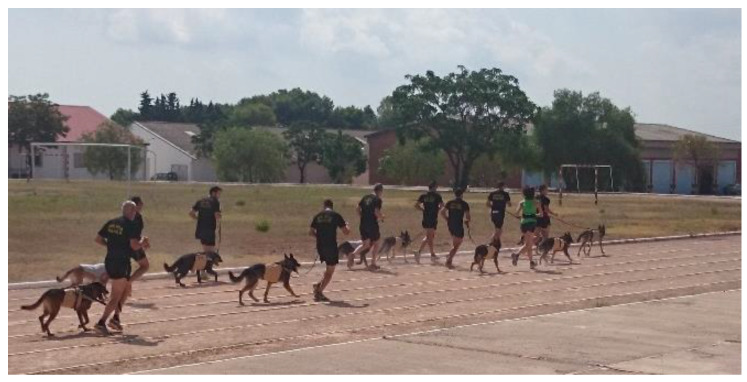
Image of one of the days of the clinical study. The circuit used in this clinical study was the one these dogs usually performed, on land, with a total duration of 20 min and at an average speed of 10 km/h, reaching 3.43 km.

The field test consisted of a routine training exercise for these MWDs and consisted of 20 min of running at a pace of 10 km/h, together with their canine guides (Figure 1), in the usual facilities of this k-9 unit (Valencia, Spain). Each handler recorded the total activity (route, running pace and distance) using a Garmin^®^ Forerunner 45 GPS watch (Garmin International, Olathe, KS, USA & Garmin Corporation, New Taipei City 221, Taiwan, R.O.C.) with heart rate monitor over the three days of the study. Environmental conditions of temperature and relative humidity were recorded using a wireless device (Gesa^®^ Weather Logic Station, Urduliz (Vizcaya), España), which was placed at the outdoor exercise site each day of the study.

Each dog was randomly assigned a condition for each day of the clinical study, so that all dogs passed through the three conditions: without cooling vest, with PCM-CV or with Ev-CV. During the clinical follow-up, the core temperature, systemic blood pressure and pulse rate of each dog were recorded at four times: in the kennel; immediately before the start (start line); immediately upon arrival (finish line) and 15 min after exercise (15’ post-exercise).

After completion of the run, the dogs rested in an airy place (no post-exercise activity period and no stretching).

### 2.3. Temperature Measurements

Digital electronic thermometers QUIRUMED®, Valencia, Spain, with a thermistor-type sensor were used to determine rectal temperature. The manufacturer temperature range was 89.6–111.2 °F (32.0–44.0 °C). Accuracy: ±0.2 °F 95.0–102.2 °F/±0.1 °C 35.0–39.0 °C/±0.2 °C the rest. Resolution: 0.1 ° for 0.1 °C. Conditions of use: 32–122 °F (0–50 °C) and Humidity: 10–83% [11]. The reading usually lasts 10 s after the highest temperature is reached, although in this study it was maintained for three minutes.

All thermometers were purchased for this clinical study, and, prior to their use, metrology was carried out to calibrate the instruments. Once their calibration had been performed, each handler carried a thermometer for his dog and no hygienic protective covers were used. Each guide and assistant were previously trained in the temperature measurement method.

### 2.4. Blood Pressure and Pulse Rate Measurements

**Figure 2:**
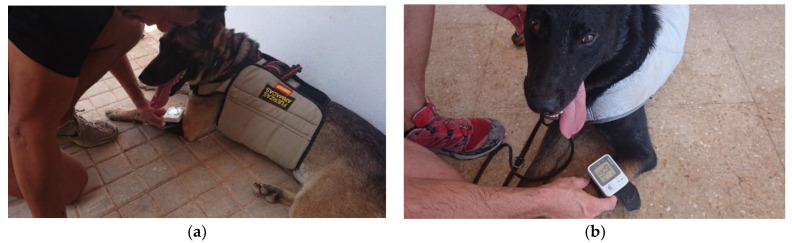
Non-invasive systemic blood pressure measurement in dogs immediately after exercise (**a**) with PCM-Cooling vest (by contact with cold tablets) and (**b**) evaporation cooling vest.

Systemic arterial pressure was determined non-invasively, with an oscillometric method, using an OMRON© HEALTHCARE Co., Ltd. (Muko, Kyoto, Japan), model RS3 (HEM-6130-E) digital wrist blood pressure monitor (Figure 2). This device supports a pressure range of 0 to 299 mmHg, with a pressure accuracy of ±3 mmHg. The approximate cuff circumference of this pressure monitor is 13.5 to 21.5 cm, and it has an operating temperature and humidity of +10 to +40 °C and 30 to 85% RH, respectively [12]. Pulse rate (PR) was obtained manually, in the femoral artery. These devices were acquired for the exclusive use of this clinical study, for which a prior control of the sphygmomanometers was carried out and users’ guidelines were given to the canine handlers and assistants responsible for taking each measurement. In all cases, the left forelimb was used, immediately next to the elbow, which is the area of best adaptation to the size of the sphygmomanometer cuff.

### 2.5. Cooling Vest

#### 2.5.1. Phase Change Material Cooling Vest (PCM-CV)

**Figure 3:**
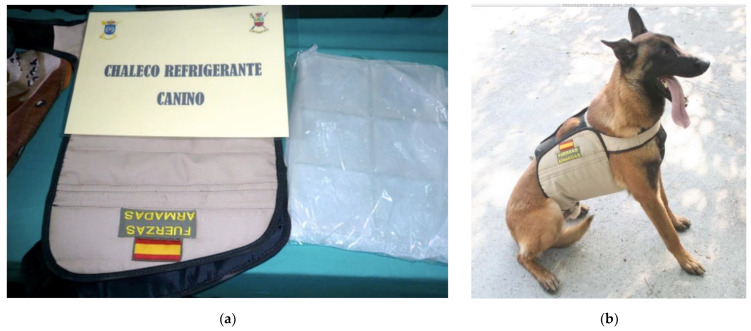
(**a**) Close-up of cooling vest (PCM-CV) with cooling pads. (**b**) An MWD wearing a PCM-CV. They require prior cooling (or freezing) of the pads.

Figure 3 shows one of the two cooling vests in the study (PCM-CV), inside which there are rigid tablets, previously refrigerated or frozen. This vest acts mainly on the cranial half of the dog’s trunk (lateral and ventral side), and the maximum duration of action, once in place, is 4 h. The weight of the cooling vest (sizes L/XL), with the cooling pack, is 1270 g (2.8 Lbs.). To activate this cooling vest, it can be placed in the freezer for a minimum of 12 min or in the refrigerator for at least 15 min.

#### 2.5.2. Evaporative Cooling Vest (Ev-CV)

**Figure 4:**
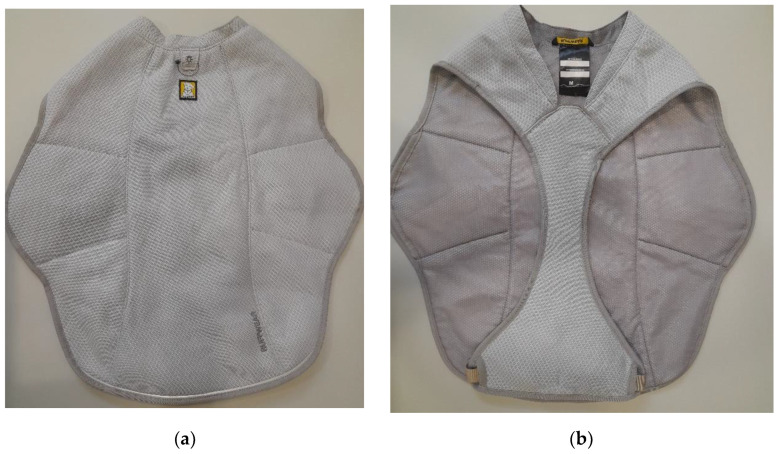
Detail of the outside (**a**) and inside (**b**) of the evaporative cooling vest Swamp Cooler^TM^ used in this clinical study. It consists of three layers to absorb water and facilitate evaporation. Requires wetting with clean water before use.

The following figure (Figure 4) shows the second model of cooling gear, used in this study, based on evaporation (Swamp Cooler© RUFFWEAR, 2843 NW, Bend, OR, USA. This cooling vest uses three layers of cloth to absorb water and facilitate evaporation. The three-layer consist of air mesh fabric made of polyester ripstop, Shinwon royal felt to hold the water, and a 100% polyester mesh lining that is in contact with the dog. It is thanks to evaporation that the dog’s surface temperature cools down [13]. This vest is wrapped around the dog and acts on the entire trunk, both in the dorsal and central areas and requires 2–5 min for activation. The weight of this cooling vest for dogs (size XL) is 380 g (0.8 Lbs.) when dry, rising to 780 g (1.7 Lbs.) when moistened for use.

### 2.6. Ambient Conditions

**Figure 5:**
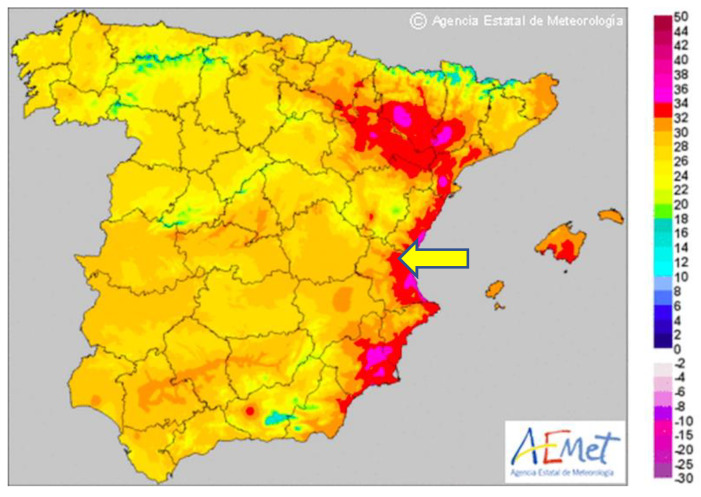
This map shows the maximum temperatures on one of the three days of the clinical study (the arrow indicates the province of Valencia (Spain), where this clinical study was carried out) (Source: Spanish Meteorology Agency AEMET).

In Figure 5, the yellow arrow shows the study area, Valencia on the map of Spain. Considered a BSk climate on the Köppen−Geiger scale, the climate of the study area, where these dogs are housed and trained, is a warm, sunny, and dry local steppe climate. This clinical study was conducted during the month of August, when the average temperature is 30 °C, whereas the average annual temperature does not exceed 18 °C (17.6 °C) [14,15].

### 2.7. Statistical Analysis

The data were analyzed with IBM SPSS Statistics for Windows, Version 27.0 (Armonk, NY, USA) IBM Corp to perform the following analyses:

Descriptive analyses. In this section, descriptive statistics were used to characterize the sample, in terms of mean age and sex.

Comparative analysis. Initially, a one-way ANOVA was performed to assess whether there were initial temperature differences in the dogs in the kennel, depending on the day of data collection.

Subsequently, two-way repeated measures ANOVA test was carried out: CONDITION of cooling with three levels (no-vest, PCM-CV and Ev-CV) and MOMENT of measurement with four levels (in kennels, at start, at arrival and 15 min after exercise), measuring core temperature, blood pressure and pulse rate in the dogs, always with the vest on in the conditions that implied its use.

## 3. Results

### 3.1. Environmental Conditions of the Field Test

Table 2 shows the characteristics of the physical exercise performed by the dogs and the meteorological parameters of the environment during the study period.

### 3.2. Initial Core Temperature of Dogs in Kennels

The initial temperature of the dogs before the start of the study corresponds to the animals’ resting temperature and was the starting point before the start of the study. Table 3 shows the average temperature of the dogs in the kennel on each of the three days of the study.

Although day 1 was the warmest day, the onset temperature of the dogs was similar on all three days, with no statistically significant differences (F = 0.073; *p* = 0.930). Therefore, the effects that may arise in relation to changes in body temperature can be attributed to CONDITION and MOMENT factors and not due to environmental temperature conditions.

### 3.3. Changes in Core Temperature Resulting from the Use or Non-Use of a Cooling Vest

A two-way repeated measures ANOVA 3 × 4 test was carried out, measuring the existence of temperature differences. The means and standard deviations in central temperature are shown in Table 4, where we observed that the maximum temperature was 41.22 °C in the “without vest” condition, at the time of arrival. All dogs lowered their temperature 15 min after the end of the test. To know if these differences are due to the CONDITION factor, we performed a variance analysis.

First, we checked that the assumption of sphericity was met, using Mauchly’s test. In the analysis of the CONDITION factor and the CONDITION * MOMENT interaction, we accepted the null hypothesis, which leads us to believe that there is no violation of the assumption of sphericity and that the analysis of variance is adequate.

Given that, for the MOMENT factor, the assumption of sphericity is not met, the Greenhouse–Geisser test is probably the most appropriate value to use, although if we have relatively few participants it may tend to be too conservative (i.e., its use will decrease the chances of finding a significant result). You can see Mauchly’s test in Table 5.

Thus, we obtained the following values for the factor CONDITION F (2, 24) = 6.927; *p* = 0.004; Partial Eta squared = 0.366. For the MOMENT factor we observed an F (1.2, 15.31) = 77.98; *p* < 0.001; partial Eta squared = 0.867. Finally, for the CONDITION × MOMENT interaction we obtained an F (6, 72) = 2.681; *p* = 0.021 and a partial Eta squared of 0.183.

We thus observed significant differences in each of the two factors as well as in the interaction between them with respect to the dependent variable body temperature. The partial Eta squared value provided us with the percentage of variance explained by each of the analyzed factors or by their interaction. Therefore, the factor that explained the greatest variance by itself was the moment, with 87% of the variance due to the factor itself.

Since the factors had more than two levels and showed significant differences, we proceeded to analyze the direction of these differences and between which levels they occured, applying the Bonferroni test (Table 6).

Table 6 shows significant differences for the condition factor *p* = 0.012; I−J = 0.427 between not wearing a vest and using Ev-CV, in the sense that there was a significantly lower body temperature when the dogs were wearing the vest.

Regarding the moment of temperature measurement, there were differences between all possible combinations, with the temperature always being lowest when the dogs were in the kennel and highest after exercise, as shown in Table 7.

Without considering the CONDITION factor, we clearly observed, as expected, that temperatures increased with physical exercise, and these differences were for all the possibilities. We also observed that after fifteen minutes of recovery, the temperature decreased, to the point of being significantly lower than the temperature obtained at the finish line. The effect of wearing or not wearing a cooling vest will have to be determined to determine whether the interaction between cooling vest and momentum generates other differences.

Isolating the MOMENT, the analysis of the interaction effects showed significant differences at the time of arrival between not wearing a cooling vest and wearing the Ev-CV (*p* = 0.011; I−J = 0.623). Lower temperatures were registered in dogs using the Ev-CV and 15 min after exercise between not wearing a cooling vest and wearing the PCM-CV (*p* = 0.015; I−J = 0.492) and not wearing a cooling vest and using the Ev-CV (*p* = 0.008; I−J = 0.769). In both interactions (Table 8), on arrival, temperatures were always lower when wearing either of the two cooling vests versus no vest.

When we analyzed the interactions between CONDITION and MOMENT, we observed (Table 9) the greatest number of significant differences occur. Thus when crossing the condition “without cooling vest”, we saw differences between all interactions except between taking the temperature without cooling vest in the kennel and taking the temperature without cooling vest at the start. The other five possible combinations showed the effects of exercise on temperature.

The same applies for the PCM-CV condition. No significant differences were found in the rectal temperature of the dogs that wore the PCM-CV in the kennel in relation to the moment of starting the race. Finally, in the condition of wearing the Ev-CV, significant differences occured in all six possible interactions.

### 3.4. Changes in Systemic Blood Pressure Resulting from the Use or Non-Use of a Cooling Vest

A two-way repeated measures ANOVA 3 × 4 test was carried out, measuring the existence of differences in systolic, diastolic, and mean arterial blood pressure. Table 10 shows the average systolic, diastolic and mean arterial blood pressure, respectively.

In the case of systolic blood pressure, the assumption of sphericity was assumed for the CONDITION factor and for the interaction between factors, while no sphericity was assumed for the MOMENT factor. Thus, the corresponding F-values were: CONDITION F (2, 24) = 0.362; *p* = 0.700; MOMENT F (1.66, 19.87) = 1.092; *p* = 0.139; CONDITION × MOMENT F (6, 72) = 1.637; *p* = 0.150.

As for diastolic blood pressure, the assumption of sphericity was assumed in all circumstances, obtaining the following F-values: CONDITION F (2, 24) = 1.285; *p* = 0.295; MOMENT F (3, 36) = 1.953; *p* = 0.139; CONDITION × MOMENT F (6, 72) = 0.893; *p* = 0.505.

The F-values obtained for the mean arterial blood pressure, assuming sphericity were: CONDITION F (2, 24) = 0.490; *p* = 0.618; MOMENT F (3, 36) = 1.722; *p* = 0.180; CONDITION × MOMENT F (6, 72) = 1.000; *p* = 0.432.

As can be seen, in the case of systolic, diastolic and mean arterial blood pressure, there was no significance in any of the analyses we carried out. It seems that for this type of exercise (intensity and duration), blood pressure was not affected by any of the analyzed factors (condition and time) or their interaction.

### 3.5. Changes in Pulse Rate Resulting from Use or Non-Use of Cooling Vest

A two-way repeated measures ANOVA 3 × 4 test was carried out, measuring the existence of pulse rate differences. The means and standard deviations in pulse rate are shown in Table 11, where the maximum registered pulse rate was 123 beats per minute in the “no waistcoat” condition at the time of arrival. Immediately afterwards (see Table 11), we observed that all dogs lowered their pulse rate 15 min after the end of the test. As in the previous analyses, the analysis of variance allowed us to know if these differences were due to the CONDITION factor.

Once the sphericity test (Table 12) was carried out, we accepted the assumption of sphericity for the CONDITION factor and for the interaction between the two factors.

However, with respect to the MOMENT factor, the assumption of sphericity was not met, so we used the F value of the Greenhouse–Geisser test, which is the most commonly used.

Thus, we obtained the following values for the factor CONDITION F (2, 24) = 1.011; *p* = 0.379; for the factor MOMENT we have an F (1.89, 22.64) = 19.817; *p* < 0.001; partial Eta squared = 0.623. Finally, for the interaction CONDITION × MOMENT we obtained an F (6, 72) = 1.284; *p* = 0.275.

In this case, there were significant differences with respect to the MOMENT factor, with 62% of the variance being explained by the effect of the time of measurement. As before, since the MOMENT factor had four levels and showed significant differences, we proceeded to analyse the direction of these differences and between which levels they occured, applying the Bonferroni test, whose results are shown below in Table 13.

With regard to the dependent variable pulse rate, significant differences were obtained for the MOMENT factor. As it had four levels, the differences between pairs were carried out, obtaining the following results.

Differences were found between the pulsations in the kennel and on arrival *p* < 0.001; I−J = −32.359. Likewise, differences were also found in the pulsations on departure versus arrival *p* = 0.004; I−J = −28.256. Finally, differences were also found between pulsations on arrival and post-exercise rate pulse *p* < 0.001 I−J = 20.487.

Finally, with regard to the effects obtained in the interaction when isolating the condition variable, the significant differences appeared, whose values are shown in Table 14.

As can be seen, the effect of the cooling vest hads its benefits, since, when the dogs wore the Ev-CV, the reduction in heart rate (pulse) at arrival and 15 min post-exertion was statistically significant, whereas the dogs wearing the PCM-CV showed no statistically significant differences in pulse rate recovery.

## 4. Discussion

The main finding of this study was that, after physical exercise in heat, the highest body temperature was registered in dogs that had not worn any cooling vest, reaching a core temperature of 41.22 °C. The variability between the two tested cooling vests (PCM-CV and Ev-CV) was very low; however, there were significant differences between the no vest and Ev-CV conditions. Thus, when the dogs were wearing Ev-CV, their mean temperature was 0.62 °C lower than the temperature of the dogs that did not wear any cooling vest. This difference, which is less than expected for humans [16], may be of great interest, since the objective of these cooling vests is not to cause a sharp drop in temperature, but rather to maintain thermal neutrality. An excess of refrigeration can cause local thermal discomfort. In addition, a temperature reduction of 0.62 °C, from a clinical point of view, reduces the risks of heat stroke. Although the safety thermal limit in dogs is unknown, it has been suggested that this limit is very close to the temperature range during exercise in other mammals [17]. Therefore, military working dogs (MWDs) may benefit from evaporative cooling vests when they need to control body temperature during moderate physical exertion in hot environments. This benefit was observed both during exercise and in the post-exercise recovery period, the greatest difference occurring 15 min post-exercise, with the dogs’ body temperature being 0.77 °C lower when using the Ev-CV than when not wearing a cooling vest. As has been demonstrated in humans [18], the use of these cooling vests is not indicated for the treatment of heat stroke or for the drastic or rapid reduction in temperature, since the method of immersion in water seems more effective [19]. This method, however, is not without risk: if the water temperature is too low, it can cause severe vasoconstriction that could lead to shock. In case of high-water temperatures, it can be counterproductive to the mechanism of heat removal by convention. Furthermore, in any case, if immersion in water is chosen, the dog should never be left unsupported, as the swimming movement itself will lead to a further increase in temperature, fatigue and collapse.

Therefore, evaporative cooling vests (Ev-Cv) are useful for the prevention of hyperthermia associated with physical exertion in hot environments which, together with a correct strategy of adequate hydration [5] and individualized monitoring of each dog [20], will result in improved welfare in scent detection dogs. These cooling vests are a better alternative to other cooling methods proven for humans, such as the use of salicylate creams [21], whose use is controversial in dogs as it is difficult to control their skin absorption, given the coat of these animals. In addition, these creams include menthol, which can produce a potential olfactory saturation effect, undesirable for an MWD’s work. With respect to PCM-CV cooling vests, the reason for their lower efficacy in this study is unknown. Since the cooling rate of these materials depends mainly on the temperature gradient between the coolant (i.e., the PCM) and the medium to be cooled (i.e., the skin), as well as the size of the cooled area, it is suggested that the extension of the vest may not envelop the entire trunk and its surface area may be insufficient. On the other hand, it is possible that the cooling power of the cooling pads was not sufficient, either because of their total mass or their latent heat of fusion [7,22].

Authors such as Hasegawa and Fullagar [23,24] demonstrated the reduction in heart rate by using cooling vests to prevent heat-induced performance alterations. However, in our study, blood pressure and heart rate did not show any dependence on central temperature and is in line with other comparative studies in humans [25]. Therefore, the condition of an MWD’s wearing cooling vest has only benefited internal temperature, as no significant differences were found in either systemic blood pressure or pulse rate in this study. It is possible that a critical threshold of core temperature must be exceeded to demonstrate cardiovascular changes in each of the study conditions.

Future research directions shall be directed to investigate the relationship between the time of use of cooling vests (prior to exercise, in active warm-up or recovery period) and canine sports performance, as has been demonstrated in humans [8]. Moreover, further research would be advisable to assess the comfort of the different cooling vests in all olfactory detection work situations.

## 5. Conclusions

The results of our study suggest that a reduction in core body temperature was achieved in dogs accustomed to training in moderately elevated temperatures with the use of cooling vests. Both types of cooling vests improved recovery of core body temperature, although evaporative cooling vests showed the best result. This allows us to recommend the use of cooling vests during physical exercise to prevent fatigue or even heat stroke. Nevertheless, no empirical evidence was found related to the use of these cooling vests and changes in blood pressure and heart rate, though the moment of the measure of heart rate affects their values. In this sense, as we anticipated, the highest pulse was observed at the finish line independently of the type of cooling vest.

## 6. Limitations of the Study

One of the main limitations of the study may be the small sample size. However, our results are very illustrative of the effect of using or not using these two types of cooling vests. Moreover, the small sample size is compensated by the homogeneity of the study group.

Therefore, given the homogeneity of the sample, and the fact that all dogs underwent the same training under the three experimental conditions, we can be optimistic that the obtained results, especially the observed statistically significant differences, are due to the experimental conditions. In any case, it is necessary to reflect on the difficulty of finding larger sample sizes.

## Figures and Tables

**Table 1:** Information on study population.

Dog Number	Breed	Age	Sex
Dog 1	GS ^1^	4	M
Dog 2	GS	3	F
Dog 3	GS	2	M
Dog 4	BSM ^2^	6	M
Dog 5	GS	4	M
Dog 6	GS	4	M
Dog 7	GS	3	M
Dog 8	GS	4	M
Dog 9	BSM	3	F
Dog 10	BSM	3	F
Dog 11	GS	6	M
Dog 12	BSM	4	M
Dog 13	GS	6	M

^1^ German Shepherd; ^2^ Belgian Shepherd Malinois.

**Table 2:** Data on environmental conditions and intensity of effort.

Title 1	Day 1	Day 2	Day 3
Distance covered (km)	3.81	3.79	3.70
Time (min:s)	21:02	21:57	20:01
Race pace (min/km)	5:31	5:48	5:25
Average speed (km/h)	10.9	10.4	11.1
Ambient temperature (°C)	31	27.8	28.4
Relative Humidity (%)	53	46	49

**Table 3:** Ambient temperature and body temperature of the dogs in kennel for each day of the trial test.

Clinical Trial Day	Ambient Temperature (°C)	Average Body Temperature of Dogs in the Kennel (°C)
Day 1	31.0	38.6
Day 2	27.8	38.5
Day 3	28.4	38.5

**Table 4:** Mean values and standard deviations for the dogs’ core body temperature (°C).

CONDITION	MOMENT	Mean	SD	N
Without Cooling Vest	Kennel	38.5692	0.82400	13
	Start line	38.8462	0.41153	13
	Finish line	41.2154	1.08769	13
	15′ post-effort	40.5923	0.78895	13
With PCM-CV ^a^	Kennel	38.5692	0.80766	13
	Start line	38.8923	0.53301	13
	Finish line	40.8231	0.60021	13
	15′ post-effort	40.1000	0.77244	13
With Ev-CV ^b^	Kennel	38.3769	0.55401	13
	Start line	38.7231	0.35626	13
	Finish line	40.5923	0.67511	13
	15′ post-effort	39.8231	0.74516	13

^a^ Phase Change Material Cooling Vest; ^b^ Evaporation Cooling Vest.

**Table 5:** Mauchly’s Test of Sphericity for the two factors and their interaction.

	W de Mauchly	Chi Square	df	*p*
CONDITION	0.911	1.023	2	0.600
MOMENT	0.058	30.491	5	0.000
CONDITION × MOMENT	0.133	19.743	20	0.500

**Table 6:** Pairwise comparisons for the CONDITION factor. Measurement: temperature.

CONDITION (I)	CONDITION (J)	Mean Difference (I−J)	Std. Error	*p* ^a^
Without CV ^b^	With PCM-CV	0.210	0.356	0.356
	With Ev-CV	0.427 *	0.012	0.012
With PCM-CV ^c^	Without CV	−0.210	0.356	0.356
	With Ev-CV	0.217	0.130	0.130
With Ev-CV ^d^	Without CV	−0.427 *	0.012	0.012
	With PCM-CV	−0.217	0.130	130

* The difference in means was significant at the 0.05 level. ^a^ Adjustment for several comparisons: Bonferroni. ^b^ Cooling Vest; ^c^ Phase Change Material Cooling Vest; ^d^ Evaporation-Cooling Vest.

**Table 7:** Pairwise comparisons for the MOMENT factor. Measure: temperature.

MOMENT (I)	MOMENT (J)	Mean Difference	Std. Error	*p* ^a^
Kennel	Start line	−0.315 *	0.071	0.005
	Finish line	−2.372 *	0.218	0.000
	15′ post-effort	−1.667 *	0.235	0.000
Start line	Kennel	0.315 *	0.071	0.005
	Finish line	−2.056 *	0.195	0.000
	15′ post-effort	−1.351 *	0.198	0.000
Finish line	Kennel	2.372 *	0.218	0.000
	Start line	2.056 *	0.195	0.000
	15′ post-effort	0.705 *	0.088	0.000
15′ post-effort	Kennel	1.667 *	0.235	0.000
	Start line	1.351 *	0.198	0.000
	Finish line	−0.705 *	088	0.000

Based on estimated marginal means. * Mean difference was significant at the 0.05 level. ^a^ Adjustment for multiple comparisons: Bonferroni.

**Table 8:** Pairwise comparisons isolating the MOMENT factor. Measurement: temperature.

MOMENT	CONDITION (I)	CONDITION (J)	Mean Difference (I−J)	Std. Error	*p* ^a^
Kennel	Without CV ^b^	PCM-CV	−7.105 × 10^−15^	0.248	1.000
		Ev-CV	0.192	0.215	1.000
	PCM-CV ^c^	Without CV	7.105 × 10^−15^	0.248	1.000
		Ev-CV	0.192	0.171	0.849
	Ev-CV ^d^	Without CV	−0.192	0.215	1.000
		PCM-CV	−0.192	0.171	0.849
Start line	Without CV	PCM-CV	−0.046	0.131	1.000
		Ev-CV	0.123	0.071	0.324
	PCM-CV	Without CV	0.046	0.131	1.000
		Ev-CV	0.169	0.117	0.524
	Ev-CV	Without CV	−0.123	0.071	0.324
		PCM-CV	−0.169	0.117	0.524
Finish line	Without CV	PCM-CV	0.392	0.219	0.297
		Ev-CV	0.623 *	0.174	0.011
	PCM-CV	Without CV	−0.392	0.219	0.297
		Ev-CV	0.231	0.122	0.246
	Ev-CV	Without CV	−0.623 *	0.174	0.011
		PCM-CV	−0.231	0.122	0.246
15′ post-effort	Without CV	PCM-CV	0.492 *	0.143	0.015
		Ev-CV	0.769 *	0.204	0.008
	PCM-CV	Without CV	−0.492 *	0.143	0.015
		Ev-CV	0.277	0.167	0.371
	Ev-CV	Without CV	−0.769 *	0.204	0.008
		PCM-CV	−0.277	0.167	0.371

Based on estimated marginal means. * Difference in means is significant at the 0.05 level. ^a^ Adjustment for multiple comparisons: Bonferroni; ^b^ Cooling Vest; ^c^ Phase Change Material Cooling Vest; ^d^ Evaporation Cooling Vest.

**Table 9:** Pairwise comparisons isolating the CONDITION factor. Measurement: temperature.

CONDITION	MOMENT (I)	MOMENT (J)	Mean Difference (I−J)	Std. Error	*p* ^a^
Without Cooling Vest		Start line	−0.277	0.153	0.570
	Kennel	Finish line	−2.646 *	0.275	0.000
		15′ post-effort	−2.023 *	0.253	0.000
		Kennel	0.277	0.153	0.570
	Start line	Finish line	−2.369 *	0.260	0.000
		15′ post-effort	−1.746 *	0.207	0.000
		Kennel	2.646 *	0.275	0.000
	Finish line	Start line	2.369 *	0.260	0.000
		15′ post-effort	0.623 *	0.148	0.007
		Kennel	2.023 *	0.253	0.000
	15′ post-effort	Start-line	1.746 *	0.207	0.000
		Finish line	−0.623 *	0.148	0.007
With PCM-CV ^b^		Start line	−0.323	0.136	0.210
	Kennel	Finish line	−2.254 *	0.295	0.000
		15′ post-effort	−1.531 *	0.341	0.004
		Kennel	0.323	0.136	0.210
	Start line	Finish line	−1.931 *	0.224	0.000
		15′ post-effort	−1.208 *	0.268	0.004
		Kennel	2.254 *	0.295	0.000
	Finish line	Start line	1.931 *	0.224	0.000
		15′ post-effort	0.723 *	0.136	0.001
		Kennel	1.531 *	0.341	0.004
	15′ post-effort	Start-line	1.208 *	0.268	0.004
		Finish line	−0.723 *	0.136	0.001
With Ev-CV ^c^		Start line	−0.346 *	0.081	0.006
	Kennel	Finish line	−2.215 *	0.182	0.000
		15′ post-effort	−1.446 *	0.247	0.000
		Kennel	0.346 *	0.081	0.006
	Start line	Finish line	−1.869 *	0.146	0.000
		15′ post-effort	−1.100 *	0.195	0.001
		Kennel	2.215 *	0.182	0.000
	Finish line	Start line	1.869 *	0.146	0.000
		15′ post-effort	0.769 *	0.163	0.003
		Kennel	1.446 *	0.247	0.000
	15′ post-effort	Start-line	1.100 *	0.195	0.001
		Finish line	−0.769 *	0.163	0.003

Based on estimated marginal means. * Mean difference is significant at the 0.05 level. ^a^ Adjusting for multiple comparisons: Bonferroni; ^b^ Phase Change Material Cooling Vest; ^c^ Evaporation Cooling Vest.

**Table 10:** Mean values and standard deviations for systolic blood pressure (N = 13).

CONDITION	MOMENT	SYSTOLIC BLOOD PRESSURE		DIASTOLIC BLOOD PRESURE		PULSE RATE	
		Mean	SD	Mean	SD	Mean	SD
Without Cooling Vest	Kennel	123.0769	32.42289	75.3077	15.16786	87.9231	16.32208
	Start line	121.6923	24.49961	76.3077	15.30460	84.4615	17.45764
	Finish line	113.5385	23.63125	71.7692	15.42808	123.3846	26.94605
	15′ post-effort	114.3077	27.85793	74.3077	20.84220	96.1538	24.67741
With PCM-CV ^a^	Kennel	111.1538	32.06204	70.3846	24.72360	78.2308	13.65744
	Start line	118.2308	28.51068	88.5385	26.27932	86.5385	12.16974
	Finish line	120.4615	28.53832	82.8462	17.27641	108.5385	16.32286
	15′ post-effort	133.1538	19.59101	88.3077	23.78860	96.3077	14.26759
With Ev-CV ^b^	Kennel	113.6154	25.30987	74.5385	13.53154	84.8462	15.42642
	Start line	126.0000	20.24434	81.1538	19.26069	92.3077	17.06060
	Finish line	125.9231	28.12905	74.6154	23.63450	116.1538	20.42403
	15′ post-effort	130.9231	24.08133	79.6923	19.85525	94.1538	21.80155

^a^ Phase Change Material Cooling Vest; ^b^ Evaporation Cooling Vest.

**Table 11:** Mean values and standard deviations for the pulse rate.

CONDITION	MOMENT	Mean	SD	N
Without Cooling Vest	Kennel	87.9231	16.32208	13
	Start line	84.4615	17.45764	13
	Finish line	123.3846	26.94605	13
	15′ post-effort	96.1538	24.67741	13
With PCM-CV ^a^	Kennel	78.2308	13.65744	13
	Start line	86.5385	12.16974	13
	Finish line	108.5385	16.32286	13
	15′ post-effort	96.3077	14.26759	13
With Ev-CV ^b^	Kennel	84.8462	15.42642	13
	Start line	92.3077	17.06060	13
	Finish line	116.1538	20.42403	13
	15′ post-effort	94.1538	21.80155	13

^a^ Phase Change Material Cooling Vest; ^b^ Evaporation Cooling Vest.

**Table 12:** Mauchly’s Test of Sphericity for the two factors and their interaction.

	Mauchly’s W	Chi Square	df	*p*
CONDITION	0.798	2.480	2	0.289
MOMENT	0.205	16.995	5	0.005
CONDITION × MOMENT	0.109	21.639	20	0.386

Design: Intersection. Intra-subjects design: CONDITION + MOMENT + CONDITION × MOMENT.

**Table 13:** Pairwise comparisons for the MOMENT factor. Measurement: pulse rate.

MOMENT (I)	MOMENT (J)	Mean Difference	Std. Error	*p* ^a^
Kennel	Start line	−4.103	3.684	1.000
	Finish line	−32.359 *	4.581	0.000
	15′ post-effort	−11.872	4.626	0.148
Start line	Kennel	4.103	3.684	1.000
	Finish line	−28.256 *	6.225	0.004
	15′ post-effort	−7.769	4.368	0.604
Finish line	Kennel	32.359 *	4.581	0.000
	Start line	28.256 *	6.225	0.004
	15′ post-effort	20.487 *	3.396	0.000
15′ post-effort	Kennel	11.872	4.626	0.148
	Start line	7.769	4.368	0.604
	Finish line	−20.487 *	3.396	0.000

Based on estimated marginal means. * Mean difference is significant at the 0.05 level. ^a^ Adjustment for multiple comparisons: Bonferroni.

**Table 14:** Pairwise comparisons isolating the CONDITION factor. Measurement: pulse rate.

CONDITION	MOMENT (I)	MOMENT (J)	Mean Difference (I−J)	Std. Error	*p* ^a^
Without Cooling Vest	Kennel	Start line	3.462	3.646	1.000
		Finish line	−35.462 *	7.831	0.004
		15′ post-effort	−8.231	8.250	1.000
	Start line	Kennel	−3.462	3.646	1.000
		Finish line	−38.923 *	8.945	0.006
		15′ post-effort	−11.692	8.057	1.000
	Finish line	Kennel	35.462 *	7.831	0.004
		Start line	38.923 *	8.945	0.006
		15′ post-effort	27.231 *	5.300	0.001
	15′ post-effort	Kennel	8.231	8.250	1.000
		Start line	11.692	8.057	1.000
		Finish line	−27.231 *	5.300	0.001
PCM-CV ^b^	Kennel	Start line	−8.308	4.251	0.446
		Finish line	−30.308 *	5.486	0.001
		15′ post-effort	−18.077	5.807	0.054
	Start line	Kennel	8.308	4.251	0.446
		Finish line	−22.000 *	6.087	0.021
		15′ post-effort	−9.769	4.660	0.348
	Finish line	Kennel	30.308 *	5.486	0.001
		Start line	22.000 *	6.087	0.021
		15′ post-effort	12.231	6.118	0.412
	15′ post-effort	Kennel	18.077	5.807	0.054
		Start line	9.769	4.660	0.348
		Finish line	−12.231	6.118	0.412
Ev-CV ^c^	Kennel	Start line	−7.462	5.898	1.000
		Finish line	−31.308 *	5.693	0.001
		15′ post-effort	−9.308	7.226	1.000
	Start line	Kennel	7.462	5.898	1.000
		Finish line	−23.846 *	7.362	0.043
		15′ post-effort	−1.846	7.412	1.000
	Finish line	Kennel	31.308 *	5.693	0.001
		Start line	23.846 *	7.362	0.043
		15′ post-effort	22.000 *	6.683	0.039
	15′ post-effort	Kennel	9.308	7226	1.000
		Start line	1.846	7.412	1.000
		Finish line	−22.000 *	6.683	0.039

Based on estimated marginal means. * Difference in means is significant at the 0.05 level. ^a^ Adjustment for multiple comparisons: Bonferroni; ^b^ Phase Change Material Cooling Vest; ^c^ Evaporation Cooling Vest.

## Data Availability

Not applicable.

## Abbreviations

MWDs—military working dogs; EDDs—explosive detection dogs; DDDs—drug detection dogs; GS—German shepherd; BSM—Belgian shepherd Malinois; PCM—phase change material; CV—cooling vest; PCM-CV—cooling vest based on phase change material; Ev-CV—evaporation cooling vest cooling; Tc—core body temperature; PR—pulse rate; MBP—mean blood pressure.
